# Development of Dengue Infection Severity Score

**DOI:** 10.1155/2013/845876

**Published:** 2013-11-12

**Authors:** Surangrat Pongpan, Apichart Wisitwong, Chamaiporn Tawichasri, Jayanton Patumanond, Sirianong Namwongprom

**Affiliations:** ^1^Program in Clinical Epidemiology, Faculty of Medicine, Chiang Mai University, Chiang Mai 50200, Thailand; ^2^Department of Occupational Medicine, Phrae Hospital, Phrae 54000, Thailand; ^3^Department of Social Medicine, Sawanpracharak Hospital, Nakorn Sawan 60000, Thailand; ^4^Clinical Epidemiology Society at Chiang Mai, Chiang Mai 50200, Thailand; ^5^Clinical Epidemiology Unit, Faculty of Medicine, Chiang Mai University, Chiang Mai 50200, Thailand; ^6^Department of Radiology, Faculty of Medicine, Chiang Mai University, Chiang Mai 50200, Thailand

## Abstract

*Objectives*. To develop a simple scoring system to predict dengue infection severity based on patient characteristics and routine clinical profiles. *Methods*. Retrospective data of children with dengue infection from 3 general hospitals in Thailand were reviewed. Dengue infection was categorized into 3 severity levels: dengue infection (DF), dengue hemorrhagic fever (DHF), and dengue shock syndrome (DSS). Coefficients of significant predictors of disease severity under ordinal regression analysis were transformed into item scores. Total scores were used to classify patients into 3 severity levels. *Results*. Significant clinical predictors of dengue infection severity were age >6 years, hepatomegaly, hematocrit ≥40%, systolic pressure <90 mmHg, white cell count >5000 /**μ**L, and platelet ≤50000 /**μ**L. The derived total scores, which ranged from 0 to 18, classified patients into 3 severity levels: DF (scores <2.5, *n* = 451, 58.1%), DHF (scores 2.5–11.5, *n* = 276, 35.5%), and DSS (scores >11.5, *n* = 50, 6.4%). The derived score correctly classified patients into their original severity levels in 60.7%. An under-estimation of 25.7% and an over-estimation of 13.5% were clinically acceptable. *Conclusions*. The derived dengue infection severity score classified patients into DF, DHF, or DSS, correctly into their original severity levels. Validation of the score should be reconfirmed before application of routine practice.

## 1. Introduction

Dengue infection has become an international public health burden. Half of the world population are presently at risk of dengue infection. Approximately 50–100 million infected cases were reported annually. Among those infected, 500000 patients had severe infection and required hospital admission; most were children. Approximately 2.5% died from the infection [1]. The cost of care was as high as $US 2.1 billion per year in The United States of America [2]. No specific treatments are available except for symptomatic [3], which are effective in early detection [4]. In patients with severe infection, shock and hemorrhage usually follow [4, 5]. If not treated, death may be a consequence. Early detection or correct prognostication may avoid such severe complications [4, 6].

Clinical risks and various laboratory results were studied to explore their roles in the prediction of dengue severity. Among many of them were girls [7], children above 5 years of age [8], persistent abdominal pain [9], lethargy, cold hand and feet [10], hepatomegaly [11], abnormal bleeding [12], overweight [13], malnourished children [14], ascites [8], plural effusion [15], leucopenia (white blood count < 4,000/*μ*L) [8], thrombocytopenia [16], hemoconcentration [16], prolonged prothrombin time (PT) [16], prolonged partial thromboplastin time (PTT) [17], elevated aspartate aminotransferase (AST), and alanine aminotransferase (ALT) enzymes [18].

The scoring system, such as the Pediatric Logistic Organ Dysfunction (PELOD) Score and the Disseminated Intravascular Coagulation (DIC) Score, was used to forecast mortality in DSS [19]. A decision tree algorithm was used to differentiate dengue fever from other types of fever and also to forecast severity in dengue infection [20]. Some studies applied DIC scoring system to diagnose DIC more precisely, using clinical signs and symptoms and routine laboratory investigations to differentiate DHF from DF [21]. Most of the prediction systems in the past focused on clinical outcomes of the disease. There are few studies that focused directly on dengue infection severity.

The aim of the present study was to develop a simple clinical risk scoring system to predict dengue infection severity, based on patient clinical characteristics and routine laboratory investigations, obtained from the previous investigation [22].

## 2. Materials and Methods

### 2.1. Patients

The medical records of children aged 1–15 years with dengue infection between 2007 to 2010 in the three university-affiliated general hospitals located in the northern region of Thailand, Sawanpracharak Hospital in Nakorn Sawan, Uttaradit Hospital in Uttaradit, and Kamphaeng Phet Hospital in Kamphaeng Phet, were reviewed. The data were retrieved from the hospital database, under the following ICD-10: A90-dengue fever, A91-dengue hemorrhagic fever, and A910-dengue hemorrhagic fever with shock.

### 2.2. Indicator Parameters

The patient characteristics with potential prediction included

1. demographic: gender and age;
2. mode of presentation: hepatomegaly, headache, myalgia, vomiting, cough, abdominal pain, rash, pleural effusion, petechiae, and any bleeding episodes;
3. hemodynamic profiles: pulse pressure, systolic blood pressure (SBP), and diastolic blood pressure (DBP);
4. hematological profiles: hematocrit, hemoglobin, white cell count, lymphocytes, neutrophils, and platelet;
5. biochemical profiles: aspartate aminotransferase (AST), alanine aminotransferase (ALT), prothrombin time (PT), and partial thromboplastin time (PTT).

### 2.3. Definition of Dengue Severity

The severity of dengue infection was operationally defined by the following criteria:

1. dengue infection (DF): acute or abrupt onset of fever, accompanied by a positive tourniquet test, and white blood count ≤ 5,000/*μ*L [23];
2. dengue hemorrhagic fever (DHF): all of the following items [24]:
  - acute or abrupt fever for 2–7 days,
  - at least one of the following bleeding episodes:
    - positive tourniquet test,
    - petechiae, ecchymoses, or purpura,
    - bleeding from mucosa, gastrointestinal tract, injection sites, or other location,
    - hematemesis or melena,
  - platelet ≤ 100,000/*μ*L,
  - at least one of the following plasma leakage evidence items:
    - hemoconcentration assessed by an increase in hematocrit ≥20% from previous hematocrit,
    - signs of plasma leakage, such as pleural effusion or ascites, or evidence of hypoalbuminemia;
3. dengue shock syndrome (DSS): all items for dengue hemorrhagic fever above, accompanied with evidence of circulatory failure [24]:
   (i) rapid and weak pulse,
   (ii) pulse pressure ≤ 20 mmHg,
4. or manifested by
   (i) hypotension,
   (ii) cold body temperature or irritable.

Dengue severity was classified into 4 grades, based on bleeding episodes and shock, as follows:

 grade 1: no evidence of bleeding, Positive Tourniquet test,
 grade 2: evidences of bleeding episodes,
 grade 3: presence of week and rapid pulse rate, low blood pressure, or narrow pulse pressure,
 grade 4: nonmeasurable blood pressure or nonpalpable pulse.
 grades 1-2 were classified as DHF and grades 3-4 were classified as DSS [24].

### 2.4. Data Analysis

Potential predictors for dengue severity from a previous report [22] were tested with nonparametric test for trend. The predictive ability was analyzed by multivariable ordinal logistic regression and presented with coefficients and odds ratios. Missing data were replaced by the mean values of the parameters. Assigned item scores were derived by transformation of coefficient of parameters. The total (sum) scores were used to classify patients into 3 severity levels. The distribution of scores across the three severity groups were presented with box plots. The discriminative and predictive abilities of the scores were presented with probability curves.

### 2.5. Ethical Approval

The present investigation was approved by the Research Ethical Committee of the Faculty of Medicine, Chiang Mai University, and the research ethical committees of the three hospitals.

## 3. Results

A total of 777 patients with dengue infection were classified based on the above criteria into 3 severity levels: DF (*n* = 391), DHF (*n* = 296), and DSS (*n* = 90).

### 3.1. Significant Predictors

Under the univariable analysis, the three severity groups were similar in gender (*P* = 0.888), age (*P* = 0.903), presence of vomiting (*P* = 0.187), cough (*P* = 0.425), rash (*P* = 0.124), and DBP (*P* = 0.084) but were difference according to the presence of hepatomegaly, headache, myalgia, abdominal pain, pleural effusion, petechiae, bleeding episodes, SBP, pulse pressure, hematocrit, hemoglobin, white cell count, lymphocytes, neutrophils, platelets, AST, ALT, PT, and PTT (Table 1).

Under the multivariable analysis, the clinical characteristics with significant predictive ability for dengue severity included age > 6 years (OR = 1.46, 95% CI = 1.12–1.91, *P* = 0.005), hepatomegaly (OR = 12.31, 95% CI = 8.84–17.15, *P* < 0.001), hematocrit ≥ 40% (OR = 1.34, 95% CI = 1.10–1.64, *P* = 0.003), SBP < 90 mmHg (OR = 1.70, 95% CI = 1.32–2.17, *P* < 0.001), white cell count > 5000/*μ*L (OR = 1.40, 95% CI = 1.13–1.75, *P* = 0.002), and platelet ≤ 50000/*μ*L (OR = 3.95, 95% CI = 3.14–4.96, *P* < 0.001). The strongest predictors were hepatomegaly (OR = 12.31) and platelet ≤ 50000/*μ*L (OR = 3.95) (Table 2).

### 3.2. The Scoring System

Transformation of significant parameter coefficients into item scores was done by division of each coefficient with the smallest coefficient of the model (0.30) and rounded up or down to the nearest 0.5 integers. The item scores ranged from 0 to 8.5, and the total score ranged from 0 to 18 (Table 2).

### 3.3. Discriminations

**Figure 1:**
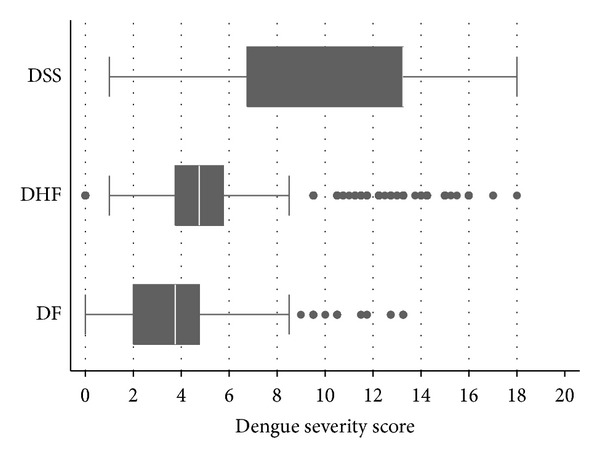
Distribution of dengue severity scores by severity levels. Vertical lines in box represent medians. Box boundaries represent the twenty-fifth and seventy-fifth percentiles.

**Figure 2:**
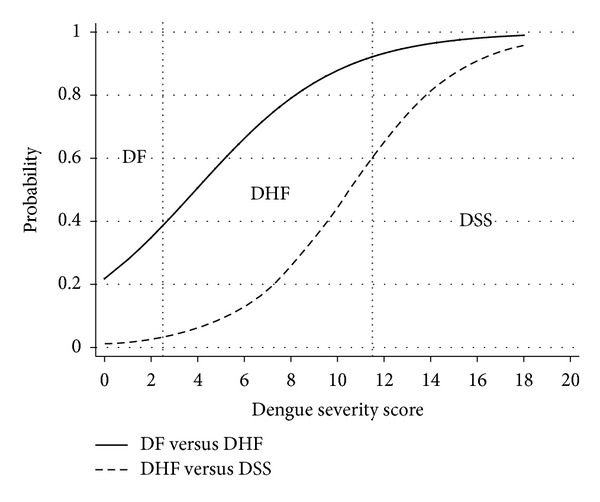
Discrimination of dengue severity scores. Solid line: dengue fever (DF) versus dengue hemorrhagic fever (DHF). Dash line: dengue hemorrhagic fever (DHF) versus dengue shock syndrome (DSS). Vertical dotted lines represent boundaries (cut-off points) of the scores.

The mean total severity scores in patients with DF, DHF, and DSS were 3.6 ± 2.1, 5.1 ± 3.2, and 11.0 ± 4.1 (Table 3). The distribution of the derived severity scores was different among the three severity groups (Figure 1). The derived scores also discriminated DHF from DF and also discriminated DSS from DHF (Figure 2).

### 3.4. Clinical Predictions

Our scoring system discriminated DSS and DHF from DF with an area under the receiver operation curve (AuROC) of 0.7416 (95% CI = 0.7294–0.7537, figure not shown) and discriminated DSS from DHF and DF with a higher AuROC of 0.8877 (95% CI = 0.8788–0.8964, figure not shown).

Cut-off points were assigned to classified patients as 3 severity groups: scores <2.5 (DF), scores 2.5–11.5 (DHF), and scores >11.5 (DSS). The score <2.5 predicted DF correctly in 297 out of 391 patients with 1-level underestimation in 149 patients (19.2%) and 2-level underestimation in 5 patients (0.6%) or a total of 19.8% underestimation.

The scores between 2.5 and 11.5 predicted DHF correctly in 136 out of 296 patients, with an underestimation in 46 patients (5.9%) and an overestimation in 94 patients (12.1%).

The scores >11.5 predicted DSS correctly in 39 out of 90 patients, with 1-level overestimation in 1 out of 11 patients (1.4%), with no 2-level overestimation (0%), or a total of 1.4% overestimation (Table 3).

## 4. Discussions

Dengue infection is an urgent condition requiring prompt diagnosis and treatment before patients enter into bleeding or shock states. Previous scoring systems trying to evaluate or forecast disease severity included the Dengue Fever Scoring System based on epidemiological information and clinical signs or symptoms, which might be useful in detecting DF very early prior to laboratory results [25]. Other scoring systems were the Pediatric Logistic Organ Dysfunction (PELOD) Score and the Pediatric Risk of Mortality III (PRISM III), used to evaluate the mortality rates [26], and the Disseminate Intravascular Coagulation (DIC) Score to diagnose DIC and to discriminate DF and DHF from other febrile illnesses [27].

Decision tree algorithms were also applied to classify dengue infections into DF, DHF grade I, DHF grade II, and DHF grade III [28]. They may also predict patients with low risk who may be discharged safely and select patients with high risk who should be admitted for close monitoring [29].

These studies used clinical symptoms and/or laboratory tests to evaluate or forecast the risks. Some studies included only adult patients [25, 29] which may or may not be relevant for children. In studies which included children, some studies used information on the first few days of admission in pediatric intensive care unit (PICU) [19]. In some studies, included DHF patients did not experience shock and may not be inferred to patients with DSS [21]. Risk prediction in many studies was based on nonroutine laboratory investigations [19, 20, 26, 28, 29]. Despite their high predictive powers, those nonroutine predictors were not available in many primary care centers. An investment on such facilities may not be feasible and may not be cost effective.

Our study developed a scoring system based on clinical risk and routine laboratory parameters and categorized patients with dengue infection into DF, DHF, or DSS.

1. Patients scoring <2.5 were classified as DF which is the mildest form. These patients normally do not require hospital admission even in the feverish stage. Patients may safely be handled as outpatients with symptomatic treatments and may be advised to observe any abnormal signs or symptoms with a follow-up appointment.
2. Patients scoring between 2.5 and 11.5 were classified as a risk group for DHF. These patients should be admitted to be closely monitored for early signs of plasma leakage, hemoconcentration, coagulopathy, thrombocytopenia.
3. Patients scoring >11.5 were classified as a risk group for DSS. These patients should also be admitted to monitor any early signs of shock.

Our scoring system predicted DSS correctly with a positive predictive value (PPV) of 88%, similar to other studies which reported a PPV of 82–95% [20, 26]. The score may be used to discriminate DSS from DF and DHF.

However, the fact that the derived scores in DF and DHF were more or less overlapping made it less likely to be used to differentiate DHF for DF. Other nonroutine predictors specifically for DHF may be required.

In case of continuous routine laboratory investigations, our score may also be used to monitor the patients risk for DSS as the disease progresses from day to day. In case of outpatients, this will help clinicians make decision when to admit the patients to hospital. Application of this scoring system into routine patient care may help reduce unnecessary admission and also reduce case facility in severe cases that are admitted based on high risk scores.

## 5. Conclusions

The derived dengue infection severity score classified patients into DF, DHF, or DSS, correctly into their original severity levels. Validation of the score should be reconfirmed before application into routine practice.

## Figures and Tables

**Table 1:** Patient profiles by types of dengue infection: dengue fever (DF), dengue hemorrhagic fever (DHF), and dengue shock syndrome (DSS).

Patient profiles	DF (*n* = 391)	DHF (*n* = 296)	DSS (*n* = 90)	*P* value*
	Mean ± SD	Mean ± SD	Mean ± SD	
Demographic				
Male (*n*, %)	185 (47.3)	153 (51.7)	38 (42.2)	0.888
Age (year)	9.5 ± 3.4	10.1 ± 3.1	9.0 ± 3.5	0.903
Mode of presentation				
Hepatomegaly (*n*, %)	8 (2.1)	26 (8.8)	55 (61.1)	<0.001
Headache (*n*, %)	228 (58.3)	145 (49.0)	35 (38.9)	<0.001
Myalgia (*n*, %)	84 (21.5)	30 (10.1)	12 (13.3)	0.001
Vomiting (*n*, %)	244 (62.4)	216 (73.0)	56 (62.2)	0.187
Cough (*n*, %)	120 (30.7)	94 (31.8)	32 (35.6)	0.425
Abdominal pain (*n*, %)	160 (40.9)	181 (61.2)	60 (66.7)	<0.001
Rash (*n*, %)	166 (42.5)	132 (44.6)	26 (28.9)	0.124
Pleural effusion (*n*, %)	0 (0)	20 (6.8)	34 (37.8)	<0.001
Petechiae (*n*, %)	27 (6.9)	26 (8.8)	14 (15.6)	0.016
Any bleeding episodes (*n*, %)	73 (18.7)	80 (27.0)	51 (56.7)	<0.001
Hemodynamic				
SBP (mmHg)	97.0 ± 9.2	96.9 ± 10.1	91.3 ± 10.3	<0.001
DBP (mmHg)	58.4 ± 7.7	58.2 ± 8.6	58.6 ± 8.4	0.084
Pulse pressure (mmHg)	33.1 ± 6.7	32.1 ± 7.6	24.0 ± 7.7	<0.001
Hematological				
Hematocrit (%)	38.8 ± 4.5	40.5 ± 4.8	42.4 ± 5.4	<0.001
Hemoglobin (g/dL)	12.7 ± 1.5	13.4 ± 1.5	14.1 ± 1.8	<0.001
White cell count (/*µ*L)	3748.0 ± 1706.5	4271.3 ± 1821.8	5479.3 ± 2063.4	<0.001
Lymphocytes (%)	44.9 ± 17.3	40.8 ± 15.7	40.0 ± 16.3	<0.001
Neutrophils (%)	44.7 ± 18.8	49.5 ± 18.1	48.9 ± 17.6	<0.001
Platelet (/*µ*L)	122693.6 ± 9763.9	81869.0 ± 7975.7	50481.5 ± 6262.9	<0.001
Biochemical				
AST (U/L)	126.7 ± 313.7	269.0 ± 457.1	558.9 ± 659.0	<0.001
ALT (U/L)	60.1 ± 216.1	95.0 ± 271.7	219.8 ± 413.2	0.001
PT (sec)	12.0 ± 1.5	12.1 ± 1.8	15.5 ± 9.5	0.012
PTT (sec)	30.4 ± 5.4	42.0 ± 10.0	44.7 ± 12.3	0.004
Case management				
Inbound referral (*n*, %)	43 (11.0)	78 (26.4)	48 (53.3)	<0.001
Discharged				
Alive (*n*, %)	391 (100.0)	296 (100.0)	88 (97.8)	1.000
Outbound referral (*n*, %)	0 (0)	0 (0)	2 (2.2)	
In hospital dead (*n*, %)	0 (0)	0 (0)	0 (0)	

*P value from nonparametric test for trend.

SD: standard deviation; SBP: systolic blood pressure; DBP: diastolic blood pressure; AST: aspartate aminotransferase; ALT: alanine aminotransferase; PT: prothrombin time; PTT: partial thromboplastin time.

**Table 2:** Significant predictors of dengue infection severity and assigned item scores.

Predictors	Category	OR	95% CI	*P* value	Coefficient*	Score
Age (year)	>6	1.46	1.12–1.91	0.005	0.38	1
	≤6		Ref			0
Hepatomegaly	Yes	12.31	8.84–17.15	<0.001	2.51	8.5
	No		Ref			0
Hematocrit (%)	≥40	1.34	1.10–1.64	0.003	0.30	1
	<40		Ref			0
SBP (mmHg)	<90	1.70	1.32–2.17	<0.001	0.53	2
	≥90		Ref			0
White cell count (/*µ*L)	>5000	1.40	1.13–1.75	0.002	0.34	1
	≤5000		Ref			0
Platelet (/*µ*L)	≤50000	3.95	3.14–4.96	<0.001	1.37	4.5
	>50000		Ref			0

*Coefficients from multivariable ordinal logistic regression. OR: odds ratio; CI: confidence interval; ref: reference category; SBP: systolic blood pressure.

**Table 3:** Severity score levels, severity levels, and risk estimation validity.

Severity score levels	Score range	Severity levels			Risk estimation validity*		
		DF (*n* = 391)	DHF (*n* = 296)	DSS (*n* = 90)	Over (%)	Correct (%)	Under (%)
Mean ± SD		3.6 ± 2.1	5.1 ± 3.2	11.0 ± 4.1			
IQR		2.0–4.8	3.8–5.8	6.8–13.3			

DF (*n* = 451)	<2.5	297	149	5	—	38.2	19.8
DHF (*n* = 276)	2.5–11.5	94	136	46	12.1	17.5	5.9
DSS (*n* = 50)	>11.5	0	11	39	1.4	5.0	—

				Total	13.5	60.7	25.7

*Percentage of total patients.

SD: standard deviation; IQR: interquartile range.
